# Effect of general anesthesia with thoracic paravertebral block on postoperative delirium in elderly patients undergoing thoracoscopic lobectomy: a randomized-controlled trial

**DOI:** 10.1186/s12871-021-01532-1

**Published:** 2022-01-03

**Authors:** Wei Wei, Xi Zheng, Yu Gu, Wenting Fu, Chunlin Tang, Yonghua Yao

**Affiliations:** Department of anesthesiology, cancer hospital and institute of Guangzhou medical university, Guangzhuou, 510000 Guangdong China

**Keywords:** Postoperative delirium, Thoracic paravertebral block, Video-assisted thoracoscopic lobectomy, Postoperative quality of recovery

## Abstract

**Background:**

Postoperative delirium (POD) is characterized by acute brain dysfunction, especially in elderly patients. Postoperative pain is an important factor in the development of delirium, and effective pain management can reduce the risk of POD. Thoracic paravertebral block (TPVB) can effectively relieve postoperative pain and inhibit the perioperative stress and inflammatory response. We investigated whether the combination of TPVB with general anesthesia reduced the occurrence of POD following thoracoscopic lobectomy.

**Methods:**

A total of 338 elderly patients, aged 65–80 years, who underwent elective surgery for video-assisted thoracoscopic lobectomy (VATS) were randomly assigned to either a patient-controlled intravenous analgesia group (PIA) or a patient-controlled paravertebral-block analgesia group (PBA). POD was evaluated using the 3-min diagnostic confusion assessment method (3D-CAM). The postoperative quality of recovery (QoR) was assessed with Chinese version of QoR-40 scale. Pain intensity was measured using the visual analog scale (VAS) score. Tumor necrosis factor-α (TNF-α) and neurofilament light (NFL) levels were determined using enzyme-linked immunosorbent assay (ELISA) kits.

**Results:**

Delirium occurred in 47 (28%) of 168 cases in the PIA group and 28 (16.5%) of 170 cases in the PBA group (RR 1.7, *p* = 0.03). PBA was also associated with a higher rate of overall recovery quality at day 7 after surgery (27.1% vs. 17.3%, *P* = 0.013) compared with PIA. The incremental change in surgery-induced TNF-α and NFL was greater in the PIA group than PBA group (*p* < 0.05).

**Conclusion:**

Thoracic paravertebral block analgesia is associated with lower incidence of postoperative delirium, probably due to its anti-neuroinflammatory effects. Furthermore, as a component of multimodal analgesia, TPVB provides not only superior analgesic but also opioid-sparing effects.

**Trial registration:**

The study was registered on the Chinese Clinical Trial Registry Center (www.chictr.org.cn; registration number: ChiCTR 2,000,033,238) on 25/05/2018.

## Background

Postoperative delirium (POD) is an acute and fluctuating disorder of the mental state with reduced awareness and disturbance of attention [1]. POD is a relative common and serious complication, which is associated with longer hospital stays, morbidity and mortality, long-term care amenities, and increased healthcare resource expenditure [2, 3]. A recent study suggested that the economic burden of postoperative delirium was as substantial as that of cardiovascular disease and diabetes, highlighting the urgent need for addressing the POD as a public health issue; however, POD is preventable in up to 40% of the affected patients [4].

Lung cancer is a common malignancy in China and is ranked first among all malignancies. Anatomic pulmonary resection is a major component of multimodal therapy according to the lung cancer guidelines [5]. Thoracic surgeries are closely related to severe pain, and unsatisfactory postoperative pain management could impede recovery and increase the risk of postoperative delirium. A recent meta-analysis showed that effective postoperative analgesia could reduce the risk of POD following hip fracture surgery [6]. In addition, observational studies have found that a higher postoperative pain score is associated with increased risk of delirium [7].

However, the precise mechanism for postoperative delirium has not been clarified explicitly, and neuroinflammation remains the main research interest [8, 9]. Animal experiments have showed that pain could activate microglial cells and cause neuroinflammation [10]. The activated microglial cells then influence the generation of dendritic spines and thus neuroplasticity, which promoted neuroinflammation [11]. Thus, perioperative pain management is important for curtailing the occurrence of delirium. Evidence suggests that blocking nerves emanating from the spinal cord (such as paravertebral block [PVB]) is associated with a decreased cognitive impairment in thoracic surgery [12].

This study aimed to compare the effects of two postoperative analgesic regimes on POD. We assumed that postoperative paravertebral analgesia could provide preferable analgesia and reduce opioid consumption postoperatively, which in turn, would decrease the incidence of POD following thoracoscopic lobectomy.

## Methods

The present study was approved by the ethical committee of Cancer hospital and institute of Guangzhou Medical University (ZN201857) and conducted following the Declaration of Helsinki. Written informed consent was obtained from the patients or their next of skin before randomization. Elderly patients aged 65–80 years undergoing video-assisted thoracic surgery (VATS) lobectomy with American anesthesiologist association (ASA) physical status classificationI-III were enrolled. Patients were excluded if they have a history of psychiatric disease; a baseline dementia or Mini-Mental State Examination (MMSE) score less than 23; body mass index greater 35 kg/m^2^; severe audio-visual impairments, or inability to speak Mandarin or Cantonese precluding communication; a surgery duration > 4 h; if they were alcohol or drug abuse; ICU admission after surgery; and contraindications to regional anesthesia. A nurse anesthetist, who was independent of data management and statistical analyses, generated random numbers (in a 1:1 ratio) with a block size of 4 using the website of www.randomization.com, and divided the patients into the patient-controlled intravenous analgesia group (PIA) and the patient-controlled paravertebral-block analgesia group (PBA). The results of randomization were sealed in sequentially numbered envelopes and stored by primary investigator until the end of the study or clinical emergency. The patients, the investigators responsible for postoperative follow up and the statisticians were all blinded to the randomization until the final statistical analyses were completed. The trial was also registered at the Chinese Clinical Trial Center (ChiCTR 2,000,033,238; Principal investigator: Yong-hua Yao).

### Anesthesia management

General anesthesia with endobronchial intubation was administered to the patients. Anesthesia was induced using sufentanil 0.2 to 0.4 μg/kg, propofol 1–2 mg/kg, and cisatracurium 0.2 mg/kg. A left- or right-sided double-lumen endotracheal tube (Shiley™ endobronchial tube accessories; Covidien, Mansfield, US) was inserted and the correct position was confirmed using a flexible fiberoptic bronchoscope. Anesthesia was maintained with sevoflurane by inhalation and remifentanil (0.1–0.3 μg/kg/min) by intravenous infusion; 0.05 mg/kg cisatracurium was administrated as intermittent IV bolus. The bispectral index (BIS) (A-2000 BISTM monitor; System rev.2.1, AspectTM Medical Systems, Inc., Mansfield, MA, USA) was used to monitor the depth of the anesthesia during the entire surgery. The sevoflurane concentration was adjusted to maintain a BIS value of 50 ± 10; 20% of the baseline values were controlled for the heart rate and blood pressure. Forced air warm blanket was used to ensure intraoperative body temperature of 36–37 °C.

The thoracic paravertebral block (TPVB) was conducted guided by an ultrasound (GE Healthcare, Vivid S70N) prior to anesthesia induction. The patient was sedated with 1 mg midazolam and 5 μg sufentanil before the TPVB procedure, meanwhile, pre-oxygenation with 100% oxygen of 6 L/min was delivered to the patient by a face mask. The patients in the PBA group were placed in a lateral decubitus position under aseptic conditions. The skin entry points were located 2.5–3 cm from the spinal processes at the T4 level. The needle tip was visualized (Contiplex D, 0.71 × 80 mm, 18G × 4 3/8, B. Braun Melsungen AG, Germany) and confirmed between the superior costotransverse ligament and the pleura under ultrasound guidance. An epidural catheter was placed into the thoracic paravertebral space through the Braun needle. An experimental bolus of 5 ml of 1% lidocaine was administered following a negative aspiration test. The patient-controlled analgesia (PCA) device was connected to the patients at the end of surgery, with 0.2% ropivacaine for PBA group and 2 μg/kg sufentanil for PIA group in a total volume of 100 ml, respectively. The device was programed to administer a background dose of 2 ml/h, as well as a bolus dose of 0.5 ml with a lockout interval of 15 min for 48 h. All patients will be given sufentanil 0.15 μg/kg when a chest tube was inserted for the sake of prophylaxis of hyperalgesia. Parecoxib 40 mg was administered if the visual analogue score (VAS, a 10 cm line where 0 indicates no pain and 10 the worst pain) of pain ranged from 3 to 5, otherwise, hydromorphone 0.008 mg/kg was administered if the VAS score was above 5 despite of patient-controlled analgesia.

### Surgical procedure

A utility incision of 5 cm length at the 4th intercostal space was chosen by the same group of surgeons, and two additional incisions of 1–2 cm length entering the 7th intercostal space in the posterior and anterior axillary lines at the diaphragm level were located. A 24-Fr chest tube was placed where exiting the anterior lower incision at the level of 7th intercostal space at the end of surgery. The chest tube was removed when no air leakage was observed for 6 h; the thoracic drainage volume for 24 h did not exceed 200 mL.

### Delirium assessment

**Fig. 1:**
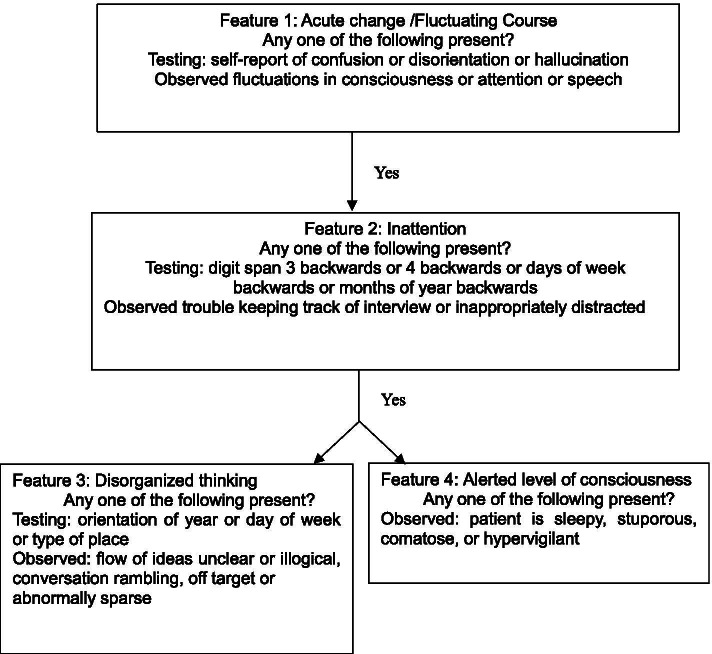
Overview of 3-min diagnostic confusion assessment method (3D-CAM) assessment. This algorithm partially refers to the published paper and obtained the permission from the copyright holder [13]

Delirium was assessed using a validated 3-min Diagnostic Confusion Assessment Method (3D-CAM, with a sensitivity of 84 to 99% and specificity of 90 to 97%) at 3 and 7 days after surgery twice daily (8:00–10:00 am) with an interval of at least 6 h. It included a 4-step algorithm and assessed, (1) acute onset of changes or fluctuations in the course of mental status, (2) inattention, (3) disorganized thinking, and (4) an altered level of consciousness. The patient was determined to be CAM positive for features 1 and 2, in addition to either feature 3 or 4 (Fig. 1) [13].

### Pain evaluation

Postoperative pain was evaluated and scored from 0 to 10, based on the visual analog scale (VAS) score by the trained clinical staff. The content of VAS was interpreted in detail to the enrolled patients at preoperative evaluation clinic. VAS score and relevant rescue analgesics were documented at 24 and 48 h postoperatively.

### Postoperative complications and recovery

Postoperative complications were evaluated for all patients enrolled in the study. We defined surgical complications as a direct result from surgery, including postoperative ventilation support (PVS), atelectasis, hemorrhage, surgical site infections (positive wound culture or antibiotics started), uncontrolled pain, delirium, pneumonia, or intensive care unit admission. Complications that were indirectly related to the surgery because of medical conditions, were defined as nonsurgical complications, including pulmonary embolism, myocardial infarction, deep vein thrombosis, fever, and other medical complications.

Postoperative recovery was assessed using the Chinese version of postoperative quality of recovery-40 (QoR-40) on the 3rd and 7th day after surgery. The QoR-40 incorporates five dimensions of health (physiology, emotion, cognition, nociception, activities of daily living); each item is graded on a five-point Likert scale. QoR-40 scores range from 40 (extremely poor.

quality of recovery) to 200 (excellent quality of recovery) [14]. Recovery was defined as a return to baseline value or better. The retain time of the chest tube and length of hospital stay were also documented.

### TNF-α and neurofilament light (NFL) levels measurement

Plasma samples were collected in the ethylene diamine tetraacetic acid-containing (EDTA) tubes and stored at − 80 °C at the following time points: pre-operation (T1), postoperative day 3 (T2), and day 7 (T3) in the morning (06:00–10:00). TNF-α measurement was conducted using enzyme-linked immunosorbent assay (ELISA), in addition to the NFL measurement using a single-molecule array method, as described [15].

### Statistical analysis

All data were analyzed using SPSS (version 22.0 for Windows; IBM Corporation, Armonk, NY, USA) and GraphPad Prism (version 5.03, GraphPad Prism Software, San Diego, California, USA). Quantitative data are expressed as the mean ± standard deviation (SD), compared with the Student *t*-test (normal distribution), or median with interquartile range (IQR) compared with the Mann–Whitney *U* test (non-normal distribution). Differences and 95% confidence intervals (CI) between medians were calculated with Hodges-Lehmann estimators. Categorical data are expressed as the number of patients (%) and were compared using the Chi-square test, the Fisher’s exact test or Kruskal-Wallis test for differences in probabilities. Relative risk (RR) and 95% CI for proportions were calculated. Repeated analysis of variance test with Bonferroni correction was applied for comparisons among the concentrations of TNFα and NFL at different time points. The statistical significance was set at *p* < 0.05.

### Sample size

Assuming a delirium incidence of 23% in PIA group and a 10% reduction in PBA group [16], 167 patients were required for each group (total of 334 patients) to show a difference in the incidence of delirium at a 0.05 significance level with a power of 0.8. With an estimated 10% attrition rate, a final sample size of 368 patients was set for the study. Due to unpredicted changes of surgical type, such as transfer to thoracotomy and palliative surgery, 338 patients were enrolled finally in the study.

## Results

**Fig. 2:**
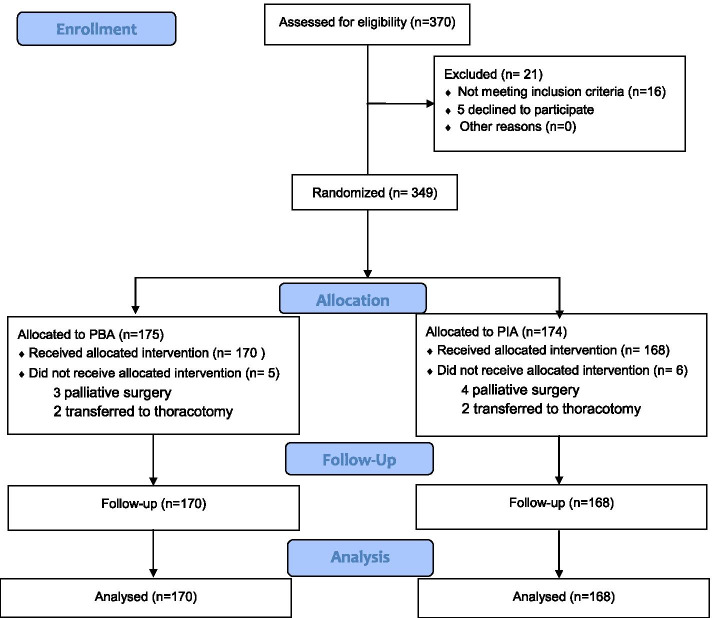
Flowchart of patient enrollment and randomization. Patients in Group PBA underwent postoperative paravertebral block analgesia; patients in Group PIA underwent postoperative intravenous analgesia. PIA, patient-controlled intravenous analgesia; PBA, patient-controlled paravertebral-block analgesia.

From April 2018 to December 2020, a total of 370 patients were assessed for their eligibility in this study. Twenty-one patients were withdrawn for not meeting the inclusion criteria (*n* = 16) or for declining consent (*n* = 5). From the remaining 349 patients, 11 were excluded as they were transferred to thoracotomy (*n* = 4) or palliative surgery (*n* = 7) intraoperatively. Finally, 338 patients were randomly assigned to either the PIA group (*n* = 168) or the PBA group (*n* = 170). A flow chart of patient enrollment is provided in Fig. 2.

The demographic data and intraoperative profiles for the two groups were comparable (Tables 1 and 2). Overall POD was detected in 75/338 (22.1%) patients, with 47/168 (28%) in the PIA group and 28/170 (16.5%) in the PBA group: relative risk 1.7, 95%CI (1.29 to 1.93), *p* = 0.03. The incidence of complications was comparable between the two groups, both the surgical and nonsurgical complications. The postoperative complications are stated in Table 3.

**Table 1:** Demographic Characteristics of the Participating Patients

Variables	PIA (*n* = 168)	PBA (*n* = 170)	*P-*value
Age (year)	73.5 ± 7.1	76.2 ± 6.3	0.471
Sex (M/F)	80/88	83/87	0.229
BMI (kg/m^2^)	24.4 ± 3.6	24.9 ± 4.0	0.337
ASA classification			0.227
II	137	146	
III	31	34	
Hypertension	77 (45.8%)	81 (47.6%)	0.802
Diabetes mellitus	39 (23.2)	36 (21.2)	0.830
COPD	17 (10.1%)	20 (11.8%)	0.567
Smoking	82 (48.8%)	96 (56.4%)	0.397
MMSE	26 [24–28]	26 [24–28]	0.920
TNM classification			0.619
I	72	76	
II	62	57	
III	34	37	

Values are expressed as mean ± SD, median [IQR] or number (percentage) when appropriate

*Abbreviations*: *BMI* Body mass index, *ASA* American Society of Anesthesiologists, *COPD* Chronic Obstructive Pulmonary Disease, *MMSE* Mini-Mental State Examination, *TNM* Tumor Node Metastasis

**Table 2:** Intraoperative and postoperative profiles

Variables	PIA (*n* = 168)	PBA (*n* = 170)	MD	95% CI	*P*-Value
Surgery time (min)	113 ± 17.1	116 ± 18.8	−2.01	− 22.13 to 20.42	0.410
Anesthesia time (min)	133 ± 19.8	141 ± 20.6	−7.94	−17.36 to 11.51	0.384
OLV time (min)	96.5 ± 14.6	98.6 ± 12.8	−2.12	−11.43 to 18.46	0.259
Blood loss (ml)	44.6 ± 5.2	43.8 ± 3.0	0.82	0.44 to1.32	0.183
Fluid balance (ml)	1670 ± 412	1741 ± 427	−71.09	− 189.3 to 57.5	0.655
Remifentanil (mg)	2.3 ± 0.6	2.2 ± 0.5	0.102	−0.17 to 0.31	0.495
Sufentanil (μg)					
Intro-operative (μg)	44 ± 3	42 ± 2	1.710	−0.75 to 2.27	0.403
Post-operative (μg)	128.8 ± 21.6	0	128.6	128.6 to 139.7	< 0.001
Parecoxib (mg)	80 [40,120]	40 [40,80]	40	20 to 80	< 0.001
Hydromorphone (mg)	0.456 [0.416–0.648]	0.184 [0.08–0.336]	0.275	0.230 to 0.315	< 0.001

Values are expressed as mean ± standard deviation, median [IQR] or number (percentage) when appropriate

*Abbreviations*: *MD* Mean difference, *OLV* One-lung ventilation

**Table 3:** Comparison of complications between the two study groups

	PIA (*n* = 168)	PBA (*n* = 170)	RR	95% CI	*P*-value
Delirium	47 (28%)	28 (16.5%)	1.70	1.29 to 1.93	0.030
3rd day incidence	21 (12.5%)	15 (8.8%)	1.42	1.22 to 1.71	0.034
7th day incidence	26 (15.5%)	13 (7.7%)	2.01	1.48 to 2.46	0.012
PVS	5 (3%)	4 (2.4%)	1.25	0.94 to 1.27	0.730
Atelectasis	6 (3.6%)	6 (3.5%)	1.02	0.76 to 1.19	0.065
Hemorrhage	1 (0.6%)	1 (0.6%)	1.00	0.42 to 1.12	0.865
Pneumonia	3 (1.8%)	2 (1.2%)	1.5	0.51 to 1.98	0.520
Incision infection	2 (1.2%)	2 (1.2%)	1.00	0.56 to 1.14	0.225
DVT	5 (3%)	3 (1.8%)	1.67	0.89 to 2.13	0.445
Ileus	11 (6.5%)	7 (4.1%)	1.59	0.94 to 2.18	0.976
AF	10 (6%)	8 (4.7%)	1.28	0.75 to 1.81	0.617

All comparisons were not statistically significant (*P* > 0.05), except for delirium (*P* < 0.05)

*Abbreviations*: *RR* Relative risk, *PVS* Postoperative ventilation support, *DVT* Deep vein thrombosis, *AF* Atrial fibrillation

The VAS scores in the PBA group were significantly lower at 24 and 48 h postoperatively compared to those in the PIA group (1.51 ± 0.40 vs. 4.13 ± 0.65, *p* < 0.001 and 1.70 ± 0.62 vs. 3.58 ± 0.49, *p* = 0.033, respectively) (Table 4). The postoperative requirement for rescue analgesics in PBA group was significantly lower than in PIA group: median difference for parecoxib 40, 95%CI (20 to 80 mg), *p* < 0.001; median difference for hydromorphone 0.275, 95%CI (0.230 to 0.315 mg), *p* < 0.001, respectively (Table 2).

**Table 4:** Visual analogue scale pain scores at 24 and 48 h after surgery in each group

	PIA (*n* = 168)	PBA (*n* = 170)	*P*-value
VAS pain score at 24th hour after surgery	4.13 ± 0.65	1.51 ± 0.40	< 0.001
VAS pain score at 48th hour after surgery	3.58 ± 0.49	1.70 ± 0.62	0.033

Values are expressed as mean ± standard deviation.

*Abbreviations*: *VAS* Visual analogue scale

The percentage of patients exhibiting overall recovery rates and recovery rates by different dimensions of QoR-40 scale at days 3 and 7 postoperatively are shown in Table 5. Compared to the PIA group, the PBA group had a higher overall recovery rate at day 7 after surgery (17.3% vs. 27.1%, *P* = 0.013). Regarding the different dimensions of QoR-40 scale, a significantly higher recovery rate was observed in PBA-treated patients for nociception, cognition and daily activities at 3 days after surgery, and for physiology, cognition, and daily activities at 7 days after surgery compared to the PIA-treated patients.

**Table 5:** Overall recovery rates and recovery rates by different dimensions of QoR-40 scale at day 3 and 7 postoperatively

	PIA (*n* = 168)	PBA (*n* = 170)	*P*-value
Postoperative day 3			
Overall recovery	3 (1.8%)	4 (2.4%)	0.751
Physiology	44 (27.9%)	52 (30.6%)	0.260
Nociception	42 (25%)	65 (38.2%)	0.013
Emotion	56 (33.3%)	78 (45.9%)	0.027
Cognition	117 (69.6%)	139 (81.8%)	0.011
Activities of day living	39 (23.2%)	67 (39.4%)	0.026
Postoperative day 7			
Overall recovery	29 (17.3%)	46 (27.1%)	0.038
Physiology	89 (52.9%)	104 (61.2%)	0.021
Nociception	134 (79.8%)	151 (88.8%)	0.454
Emotion	144 (85.7%)	152 (89.4%)	0.172
Cognition	140 (83.3%)	156 (91.8%)	0.019
Activities of day living	122 (72.6%)	135 (79.4%)	0.043
DCTP (day)	4.53 ± 1.71	3.72 ± 1.44	0.042
LOS (day)	6.57 ± 2.21	5.92 ± 2.03	0.054

Values are presented as number (percentage) or mean ± standard deviation when appropriate

*Abbreviations*: *LOS* Length of hospital stays, *DCTP* Duration of chest tube placement

**Fig. 3:**
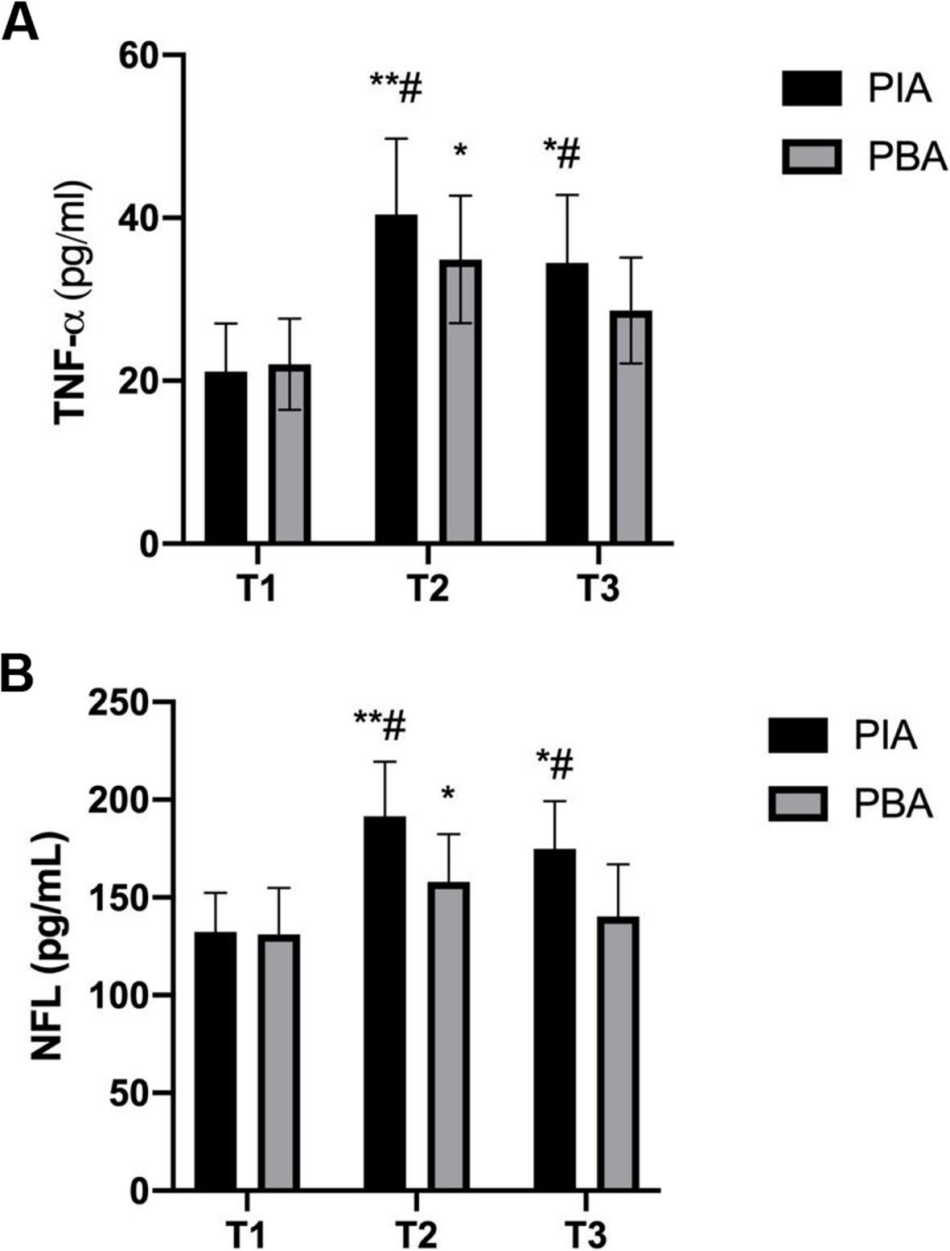
Plasma levels of S-100β (**A**) and neurofilament light (NFL) (**B**) before and after surgery between the PIA and PBA groups. ^*^ *p* < 0.05, ^**^ *p* < 0.01 vs. baseline, ^#^ *p* < 0.05 vs. PBA group. T1, before surgery; T2, 3 days after surgery; T3, 7 days following surgery. PIA, patient-controlled intravenous analgesia; PBA, patient-controlled paravertebral-block analgesia

Regarding the levels of TNF-α and NFL, both were lower in the PBA group compared to PIA group at T2 and T3 (*p* < 0.05, Fig. 3A, B).

Paravertebral block analgesia was associated with earlier chest tube withdrawn (4.53 ± 1.71 vs. 5.92 ± 2.03 days, *p* < 0.01, Table 5); However, no significant differences were found in the length of hospital stay.

## Discussion

This prospective study shows that the incidence of POD was significantly.

decreased with PBA compared to PIA postoperatively. To our knowledge, this is the first study with a large sample size to evaluate the effects of PBA regime on postoperative delirium in the elderly patients undergoing VATS lobectomy.

The development of POD lies in the complicated interaction of multiple risk factors [17]. Of note, the risks that can be modified are expectant targets for preventing POD. Strategies including the effectiveness of pain management and the minimization of opioid consumption are very promising in reducing the incidence of POD. As a component of multimodal analgesia, paravertebral block analgesia exhibited not only preferable analgesic but also opioid-sparing effects, both of which could alleviate POD. Pain and neuroinflammation triggered by pain itself could contribute to the development of POD and vice versa [7, 10]. Accordingly, our study has shown that the pain severity as well as the incidence of POD were significantly lower in PBA group. Conversely, the use of opioids (long-acting opioids in particular) is highly pertinent to the development of postoperative delirium in a dose-dependent manner [18]. Hence, it is critical to minimize the opioid consumption for curtailing POD [1, 8]. The evidence that non-opioid analgesic strategy dramatically reduced POD compared with opioid-only analgesic regimes is rising [19, 20]. Regional anesthesia technique probably played an important role in preventing POD, while both fascia iliac block and femoral nerve block prophylaxis decreased the incidence of POD in elderly patients undergoing total knee or hip arthroplasty [21, 22]. Thoracic paravertebral block (TPVB) offered adequate pain control as it effectively blocked the neural afferents, thus reducing postoperative acute pain, opioid consumption and neurocognitive dysfunction [23]. The current study also confirmed that paravertebral block analgesia was superior to intravenous analgesia in reducing the incidence of POD, indicating that opioid-sparing effect might play an indispensable role. However, a randomized, blinded trial evaluated the impact of different postoperative pain management on POD in patients undergoing transapical aortic valve replacement. The results showed that the TPVB strategy had an opioid-sparing effect, but the incidence of POD did not decrease significantly, likely due to small sample size and fewer elderly patients involved [24].

Thoracic surgery using the one-lung ventilation (OLV) technique also contributes to the development of postoperative delirium in elderly patients, with a POD incidence rate of 18.8% [17, 25]. Theoretically, OLV initiates pathophysiological changes, specifically including hypoxic pulmonary vasoconstriction which is associated with severe oxidative stress and generation of free radicals, and finally promotes the neuroinflammatory responses and the development of POD in elderly patients [26]. More importantly, surgical stress and ensuing inflammatory response can degrade the glycocalyx substances of vascular endothelial cells and increase the permeability of blood-brain barrier, thus facilitating the entry of inflammatory mediators to central nerve system [12, 27–29]. Therefore, the complicated interaction of above risk factors (pain, OLV, stress and inflammatory response) will finally promote neuroinflammation and thus exacerbate postoperative delirium.

Thoracic paravertebral block (TPVB) can intercept the sympathetic nerve conduction, and suppress the nociceptive stress and inflammatory response, especially neuroinflammation. The proinflammatory factors induced by activation of microglia cells played a critical role in neuropathological development [30, 31]. Xie et al. found that TPVB with general anesthesia could improve early postoperative cognitive function in elderly patients undergoing thoracoscopic surgery owing to its anti-inflammatory effect [32]. In response to neuroinflammation, the microglial cells release TNF-α, of which the concentration depends on the integrity of the blood-brain barrier and severity of neuroinflammation [33]. Given the correlation between neuroinflammation and postoperative delirium, we took TNF-α as an inflammatory marker to elucidate the plausible mechanism. Consistent with our assumption, the patients in the PBA group exhibited lower incidence of delirium and level of TNF-α, indicating that paravertebral block analgesia could alleviate the inflammatory response.

Besides, studies also revealed that surgery as a trigger might lead to delirium through the pathogenesis of neuroinflammation-induced neuron injury [34, 35]. As proved by Casey et al. [34], neuronal injury contributed to the pathogenesis of POD, which was accompanied with the increase in NFL level [32]. Coincidentally, we detected the concentration of NFL in the PBA group were dramatically lower compared to the PIA group after surgery, in line with the trend of TNF-α. It is plausible that paravertebral block may possess.

neuroprotective effects, but further animal and human studies are warranted.

Our study has several limitations. Firstly, although we have attempted to evaluate postoperative delirium on postoperative days 3 and 7, unidentified delirious status, especially hypoactive subtype, is still an issue because of the waxing and waning nature of delirium. Therefore, the chart-based delirium identification instrument with the information primarily derived from electronic medical records system and recalling descriptions of caregivers should be employed to detect any cases of delirium in patients that might occur outside of in-person delirium assessments. Secondly, despite its subjective and disputed role, we employed 3D-CAM as a screening tool, which is still a primary and practical diagnosis method. Delirium rating scale (DRS) may be more objective when delirium severity is considered. Thirdly, postoperative delirium and postoperative neurocognitive dysfunction may co-occur in the elderly; however, the relationship between them is not clearly elucidated [36]. Further studies investigating the associations between postoperative analgesic strategy and long-term cognition or dementia are warranted. Fourthly, previous studies confirmed the hypothesis that paravertebral block analgesia possessed anti-inflammation effect, but whether it played a role in the neuroprotective effect deserves further studies. Fifthly, absence of epidural block group is another limitation. The results of a Cochrane systematic review, which compared thoracic epidural block (TEB) and paravertebral block (PVB) on postoperative analgesic effects and complications in adults undergoing thoracotomy surgery, demonstrated that both regional blockade techniques were equally effective in controlling acute pain, while PVB is superior in reducing postoperative delirium (RR 0.3, 95%CI 0.09 to 0.99) [33]. Nevertheless, the results should be interpreted with caution due to the heterogeneity of the studies and the lack of high-level evidence, so further powered RCTs are warranted. Lastly, an additional important limitation is the combination of sufentanil with hydromorphone in the PIA group after surgery. This confounding bias reduced the reliability of study. It would been better to use the same opioid after surgery between two groups. We did not administer sufentanil as rescue analgesics after surgery in order to avoid respiratory depression in the elderly. Despite of these important limitations, our research has strengths including rigorous delirium assessment (3D-CAM with high sensitivity and specificity) and Chinese version of postoperative quality of recovery-40 (QoR-40) scale. QoR-40 scale is a multidimensional and validated survey-based tool, including a domain formulated to evaluate cognitive recovery over time, in which the recovery in various domains is defined by the values following surgery equaling or exceeding the baseline. We demonstrated that both cognitive and daily living recovery rates at 3- and 7-days after surgery were higher with paravertebral block analgesia compared with intravenous analgesia. Therefore, it is obvious that thoracic paravertebral block analgesia can accelerate postoperative recovery characterized by higher QoR-40 score, shorter retain time of chest tube and length of hospital stay. Last but not the least, each subject who received the intervention completed the follow-up process, which increased the credibility of our results.

## Conclusions

TPVB analgesia is associated with lower incidence of postoperative delirium, probably due to its anti-neuroinflammatory effects. Furthermore, as a component of multimodal analgesia, TPVB provides not only superior analgesic but also opioid-sparing effects.

## Data Availability

The datasets generated during and/or analyzed during the present study are available from the corresponding author on reasonable request.

## Abbreviations

POD: Postoperative delirium
TPVB: Thoracic paravertebral block
VATS: Video-assisted thoracoscopic lobectomy
PIA: Patient-controlled intravenous analgesia
PBA: Patient-controlled paravertebral-block analgesia
CAM: Confusion assessment method
VAS: Vsual analog scale
TNF-α: Tumor necrosis factor-α
NFL: Neurofilament light
ELISA: Enzyme-linked immunosorbent assay
OLV: One-lung ventilation
BIS: Bispectral index
3D-CAM: 3-min diagnostic confusion assessment method
PVS: Postoperative ventilation support
QoR-40: Quality of recovery-40
